# ATP11C基因新突变致先天性溶血性贫血1例

**DOI:** 10.3760/cma.j.issn.0253-2727.2022.06.016

**Published:** 2022-06

**Authors:** 慧敏 张, 柳 杨, 雯 董, 清池 刘

**Affiliations:** 河北医科大学第一医院，石家庄 050030 The First Hospital of Hebei Medical University, Shijiazhuang 050030, China

患者，男，15岁。主因“发现皮肤黄染、尿色深2个月”就诊。查体：全身皮肤及巩膜轻度黄染，无皮疹及出血点，浅表淋巴结无肿大。腹部平坦，肝脾肋缘下未触及。双下肢无水肿。血常规：WBC 5.6×10^9^/L、RBC 4.0×10^12^/L、HGB 119 g/L、PLT 261×10^9^/L，平均红细胞体积（MCV）98.1 fl、平均血红蛋白量32.0 pg、平均血红蛋白浓度（MCHC）326 g/L、网织红细胞（Ret）比例5.2％；骨髓象：增生明显活跃，粒红比为0.98∶1，粒系比例减低，红系比例增高，以中、晚幼红细胞为主，淋巴细胞比例正常，全片巨核细胞78个。血小板易见。外周血涂片红细胞无形态异常。尿常规：尿胆原17 µmol/L。肝炎病毒及自身免疫性肝炎检测均阴性。丙氨酸转氨酶、天冬氨酸转氨酶、γ-谷氨酰转肽酶正常。溶血指标：总胆红素77.6 µmol/L，间接胆红素69.3 µmol/L，直接胆红素8.3 µmol/L；乳酸脱氢酶正常；Coombs试验阴性；Flaer、CD59正常；抗碱血红蛋白、血红蛋白A2、微量血红蛋白电泳、异丙醇、红细胞渗透脆性试验、H包涵体、变性珠蛋白小体、葡萄糖-6-磷酸酶活性均正常；高铁血红蛋白还原试验：58％（参考值>75％）。血浆游离血红蛋白：37.5 mg/L（参考值<40 mg/L）；铜蓝蛋白：正常。腹部超声：肝胆脾胰肾未见明显异常。采用二代测序（NGS）对患者血样进行全外显子基因组测序，检测内容覆盖Gilbert综合征UGT1A1基因，未发现相关致病性位点的变异，检出先证者ATP11C基因存在一处半合子变异：c.1003C>T（p.R335*），Sanger测序验证显示该变异其父母均未携带，为患者新生变异。经查询千人基因组数据库、Exac数据库，该变异在东亚人群中的携带率均为0；GERP++分数为5.63，预测为有害。诊断：X连锁先天性溶血性贫血。

讨论：本例患者足月妊娠，发育正常，父母体健、非近亲结婚，无兄弟姐妹，因家属偶然发现其尿色深、面色黄首诊于消化科，排除肝病后转于血液科，排除Gilbert综合征及其他常见溶血性贫血，在X连锁先天性溶血性贫血相关基因ATP11C发现一处半合子变异而确诊。最初，日本学者对一例症状与之相似的成年男性轻度溶血性贫血患者进行全外显子测序，没有检出已知的溶血性贫血的致病基因，在X染色体上发现了ATP11C基因的错义突变（c.1253C>A，对应p.Thr418Asn），突变的先证者为半合子，母亲为杂合子，通过对ATP11C的功能分析，确定ATP11C是人红细胞膜上一种主要的翻转酶，ATP11C基因突变会干扰ATP11C的功能，导致先天性溶血性贫血。本例男性患者ATP11C基因存在一处半合子变异，家系验证为新生变异。X连锁先天性溶血性贫血罕见易漏诊，以非结合胆红素增高为主的患者，临床上除筛查常见的溶血性贫血及Gilbert综合征外，还要考虑此病，本例患者ATP11C基因突变位点c.1003C>T（p.R335*）未见报道，这可能是导致X连锁先天性溶血性贫血的一个新位点。

